# Childhood trauma, suicide risk and inflammatory phenotypes of depression: insights from monocyte gene expression

**DOI:** 10.1038/s41398-020-00979-z

**Published:** 2020-08-24

**Authors:** Carmen Schiweck, Stephan Claes, Lukas Van Oudenhove, Ginette Lafit, Thomas Vaessen, Gommaar Op de Beeck, Raf Berghmans, Annemarie Wijkhuijs, Norbert Müller, Volker Arolt, Hemmo Drexhage, Elske Vrieze

**Affiliations:** 1 Department of Neurosciences, Psychiatry Research Group, KUL University of Leuven, Herestraat 49, Leuven, Belgium; 2 Department of Psychiatry, Psychosomatics and Psychotherapy, Goethe University, Frankfurt am Main, Germany; 3 University Psychiatric Center KU Leuven, KUL University of Leuven, Leuven, Belgium; 4 Laboratory for Brain-Gut Axis Studies, Translational Research Center for Gastrointestinal Disorders, Department of Chronic Diseases, Metabolism, and Ageing, KU Leuven-University of Leuven, Leuven, Belgium; 5 Research Group of Quantitative Psychology and Individual Differences, KUL University of Leuven, Leuven, Belgium; 6 apDia, Advanced Practical Diagnostics, Turnhout, Belgium; 7 Department of Immunology, University Medical Center Rotterdam, Erasmus MC, Rotterdam, The Netherlands; 8 Department of Psychiatry and Psychotherapy, Ludwig-Maximilians-Universität, Munich, Germany; 9 Otto Creutzfeldt Center for Cognitive and Behavioural Neuroscience, University of Münster, Munich, Germany; 10 Department of Psychiatry and Psychotherapy, Münster University Hospital, Albert Schweitzer Campus 1 – A9, Münster, Germany

**Keywords:** Clinical genetics, Human behaviour, Depression

## Abstract

Circulating monocytes contribute to inflammatory processes. We here validate abnormal expression of inflammation-related genes in monocytes of a large and well-characterised group of MDD patients, and relate the outcomes to pertinent clinical characteristics. Thirty-two genes of a previously established inflammation-related gene signature were assessed in 197 patients with MDD, and 151 controls collected during the EU-MOODINFLAME project. Monocyte gene- expression data were related to age, sex, BMI, depression severity, childhood adversity (CA) and suicide risk (SR). Three distinct gene profiles were identified within the MDD group (downregulated, mixed upregulated and strongly upregulated genes). Patients in the merged upregulated groups had a significantly higher prevalence of CA and high SR. Using hierarchical clustering of the genes, we found a cluster of mainly cytokine (production)-related genes; patients with SR had a significantly higher expression of this cluster than patients without SR (particularly for IL-6, IL1A and IL1B). Such difference did not emerge for patients with and without CA. A downregulated gene profile was found for patients not exposed to CA and without SR (particularly for glucocorticoid-signalling genes NR3C1a and HSPA1/B). No inflammatory changes were observed for healthy controls exposed to CA. Our data show that inflammatory activation in MDD is not uniform, and that immunologically discernible phenotypes of depression can be linked to CA and high SR. The absence of monocyte inflammatory activation in healthy controls exposed to CA suggests an inflammatory involvement in MDD-prone individuals exposed to early stressors, but not healthy controls.

## Introduction

Major depressive disorder (MDD) is a highly prevalent disorder^1^ with heterogeneous presentation of clinical symptoms^2^. The exploration of clinical risk factors in combination with biological targets is particularly appealing for identification of MDD subgroups, but ultimately, for tailored treatment of different MDD phenotypes. One such biological target is the immune system. Over the past decades, evidence has been presented, which supports the concept that low-grade inflammation plays a role in the pathogenesis of MDD. An increase in inflammatory serum cytokines^3,4^, C-reactive protein^5^, overall leukocytosis, inflammatory monocytosis, activated glial cell status and a beneficial response to anti-inflammatory therapy have been demonstrated^6–9^. Nevertheless, there are also reports showing rises in anti-inflammatory cytokines^10,11^, reduced lymphocyte-proliferative response to mitogen^7^ and overall non-responsiveness to anti-inflammatory therapy^12^, creating a more complex picture of inflammatory activation in MDD. Recent research has suggested that high levels of inflammatory markers are associated with treatment resistance^13^, and has shown that particularly those with high levels of inflammatory markers may benefit from anti-inflammatory agents^12^. In previous research, we have contributed to the understanding of altered inflammatory states in psychopathology by studying gene expression of circulating monocytes in mood-disorder patients^14–17^. Blood monocytes are among the principal cells of the innate immune system, and take on important roles in phagocytosis, antigen presentation and cytokine production^18^. We previously established an abnormal expression pattern of inflammation-related genes (a “gene signature”), that is altered in monocytes of patients with bipolar disorder^15^, unipolar depression^14,17^, postpartum psychosis and endocrine autoimmune disorders such as diabetes^19^, pointing to a shared inflammatory activation between these disorders. The gene signature was derived by selecting the top up- or downregulated genes in pre-studies using whole-genome analyses. Additional validation was achieved in various confirmation studies using Q-PCR. The signature contains genes that have previously been implicated in the pathogensis of MDD, including interleukin (IL)-6, IL1B, TNF, the glucocorticoid receptor subunits α and β and the MAP kinase pathway. More information on the identification and validation of the signature can be found in Supplementary Notes. The consistent upregulation of genes expressed in monocytes, monocyte-derived dendritic cells and neutrophils in two separate case–control studies using blood microarrays^20^, and by others^21^, lends further independent support to the notion of aberrant activation of innate immune myeloid cells in depression

However, two important shortcomings hamper interpretability of previous findings. Firstly, it is well established that clinical characteristics, such as depression severity, suicidal ideation and childhood adversity (CA), as well as medication status, age and obesity^4,22–24^, can have an important impact on the inflammatory-response system. Yet, few current studies focus on these specific clinical characteristics. Secondly, previous studies have a limited sample size of patients or controls, which limits power to detect the additive main and interaction effects. We here tackle these caveats by exploring the relationship of inflammation-related gene expression and pertinent clinical characteristics (age, sex, BMI, depression severity, suicide risk (SR) and CA) in a large group of MDD patients.

Our primary aim for this study is to assess whether these clinical characteristics are linked to up- and/or downregulated gene expression in patients with MDD, and can explain (part of) the reported heterogeneity of inflammatory activation. For this purpose, we use in the first approach an unsupervised clustering on the well-defined, previously identified genes, to identify in an unbiased fashion subgroups of MDD patients with predominantly upregulated, downregulated or mixed-up and downregulated inflammation-related genes. We explore the distribution of clinical characteristics between these different MDD subgroups with up- or downregulated gene expression, and focussed in particular on demographic characteristcs, depression severity, CA and SR, as these characteristics have been previously linked to inflammatory activation of the immune system, as described above.

In the second approach, we performed a hierarchical clustering of the expression of the genes to identify clusters of genes correlating in expression intensity. We identified three gene clusters, and investigated whether MDD patients with or without the described clinical characteristics (e.g., CA or SR) significantly differed in the clusterwise expression of the genes. We hypothesised that upregulated inflammation-related gene expression is not uniform across patients with depression, and that depression severity, CA and SR are linked to specific inflammatory activation in MDD. In addition, as the secondary aim, we also determined serum levels of interleukin 6 (IL-6) and highly sensitive C-reactive protein (hsCRP) to assess whether monocyte gene expression is also reflected in serum markers of inflammatory activity, and whether these serum protein markers are also related to the mentioned clinical characteristics such as CA and SR.

## Materials and methods

### Participants

Data were derived from the EU-funded MOODINFLAME study (“MOODINFLAME website” 2014) carried out to investigate possible inflammatory biomarkers to advance early diagnosis, treatment and prevention of mood disorders. Participants were adults free of clinical inflammation-related symptoms, including fever and current or recent infectious or inflammatory disease, uncontrolled systemic disease, uncontrolled metabolic disease or other significant uncontrolled somatic disorders known to affect mood. Patients did not use somatic medication known to affect mood or the immune system. Pregnant or postpartum candidates were excluded. This study was set up as a cross-sectional case–control study. Peripheral blood samples of 212 controls and 202 MDD patients were analysed.

Patients were recruited from three university psychiatry clinics, i.e., Münster (Germany), which we partly reported in a previous publication^14^, and from as yet unpublished cohorts from Munich (Germany) and Leuven (Belgium). Samples were collected from 2009 to 2012 using uniform clinical selection/evaluation and laboratory techniques. The study was approved by ethical committees of the participating universities (reference numbers: Leuven: S51723, Munich: 291-09, and Münster: 2009-019-f-S), and written informed consent was obtained from all participants.

### Assessments

DSM-IV MDD diagnoses were confirmed using the mini-international neuropsychiatric interview (MINI)^25^. The severity of depression was measured by the inventory of depressive symptoms (IDS-C_30_)^26^ for patients, for controls with self-report questionnaires (IDS-SR_30_). The presence of CA was assessed with the childhood trauma questionnaire short form (CTQ-SF)^27^. Subscales of the CTQ include emotional or physical abuse, and neglect and sexual abuse. Cut-off scores to determine the presence of trauma on any subscale were previously established^28^. SR status was derived by the MINI section C, where to score high risk, patients need to endorse (1) active suicidal ideation with concrete suicidal plans, (2) have attempted suicide in the past month or (3) have attempted suicide (not during the last month) and currently actively thinking about attempting suicide. Control participants underwent psychiatric assessment with the MINI screening version. In the case of additional questions, the full MINI questionnaire was administered. Assessment of depression diagnosis with the MINI occurred on the same day as the blood sampling.

### Laboratory methods

*Determination of IL-6 and hsCRP in serum:* For the IL-6 determination, pre-coated microtiter strips were incubated with standards and diluted samples (overnight, 4 °C). Unbound serum proteins were removed (washing). Next, a monoclonal biotin conjugate was added to bind directly to the antibody–antigen complex. After incubation (1 h, 37 °C), unbound biotin antibodies were removed (washing). Next, a streptavidin poly-HRP conjugate was added and strips incubated (20 min, 37 °C). After removal of the unbound conjugate, a TMB-containing solution was added to the wells and incubated (20 min, 37 °C). A blue colour developed in proportion to the amount of IL-6 bound. The reaction was stopped (0.5 M stop solution), and signals read at 450 nm.

The hsCRP assay was done according to the apDia hsCRP (REF: 740011) manual: 10× prediluted standards were diluted 1:100 and serum samples 1:1000. Microtiter strips were incubated with the diluted standards and sera (30 min, room temperature (RT)). Any unbound serum proteins were removed (washing), and a specific HRP-conjugated antibody added to detect the antibody–antigen complex. Strips were incubated (30 min, RT). After removal of the unbound conjugate, a chromogen solution containing TMB was added. During the incubation for 10 min at RT, a blue colour develops. The reaction was stopped (0.5 M stop solution), and signals read at 450 nm.

### Monocyte gene expression

To detect expression of inflammation-related genes in CD14 + monocytes, collection, purification and determination methods were used as described in previous publications^14,17^. Shortly, RNA was isolated from CD14 + purified monocytes; to obtain cDNA for quantitative-polymerase chain reaction (q-PCR), 1 μg of RNA was reverse-transcribed using the cDNA high-capacity cDNA Reverse Transcription Kit (Applied Biosystems, Carlsbad, CA, USA). Then, relative to the housekeeping gene ABL1, the expression of the genes ADM, ATF3, BCL2A1, BTG3, CCL2, CCL20, CCL7, CD9, CDC42, CXCL2, DHRS3, DUSP2, EMP1, EREG, FABP5, HSPA1A/HSPA1B, IL-1α, IL-1β, IL1R1, IL-6, IRAK2, MAFF, MAPK6, MXD1, NAB2, PDE4B, PTGS2, PTPN7, PTX3, SERPINB2, STX1A, THBD, TNF and TNFAIP3 was determined, using the comparative threshold cycle (CT) method (Biosystems, 2001), yielding ΔCT values. Due to minor technical differences in leukocyte isolation procedures, we expressed patient data relative to values of controls of the same site by deriving ΔΔCT values before pooling data^29^. Complete control cases without CA were used as reference group. Data are deposited in the NCBI’s GEO repository (accession number GSE147582-GSE147584).

### Statistical analysis

Statistical analyses and graphical representations were performed in R version 3.5.1 and Graphpad Prism 8.0.0. Gene expression of MDD patients (*n* = 197) and controls with CA (*n* = 56) was expressed relative to complete control cases without CA (*n* = 95). Genes with a pre-established threshold of <30% of missing data per site and 20% from merged centres (*n* = 32) were used for analysis and imputed using median imputation for graphical representations, and multiple imputation using chained equations (mice package)^30^ for multivariate analysis with covariates. Predetermined power analysis yielded a necessary sample size of 196 to detect even a small group effect (i.e., Cohen’s h of 0.2 for MDD vs. HC) with 80% power.

Independent variables for the multivariate analysis were SR and childhood trauma score (both categorical), and age, BMI and depression severity (continuous) as well as sex (categorical). Comparisons for gene expression compared to healthy controls were performed using one-sample *t* tests corrected for false discovery rate (FDR) using the Benjamini–Hochberg method. To identify patient subgroups, ΔΔCT values were used for hierarchical clustering, using Euclidean distance and ward’s (ward.d2) agglomeration method^31^. Optimal numbers of clusters were determined using the package Nbclust in R^32^. Clinical characteristics of subgroups were compared for the bottom-up approach, with chi-square tests for frequencies, or Wilcoxon–Mann–Whitney test for continuous variables. We also assessed correlated gene clusters. Within these gene clusters, the effects of significant clinical variables were explored in a top-down approach. This included a comparison of patients and controls with CA or SR compared to controls without CA. Due to a relatively small sample size, *P* values are not reported with correction for FDR. Corrected values are provided in the Supplementary Information. Multivariate analysis of variance with age, sex, BMI, depression severity and CA and SR, as well as an interaction term between SR and CA, was performed on all five datasets arising from the multiple imputation. *F* values of significant effects arising during multiple imputation were pooled to calculate an approximate overall *P* value. Pooled *P* values for single genes were obtained with the mice package^30^. Separate analyses were performed adding antidepressant medication status. Contrasts for single genes are computed with the no-risk group as reference, and are reported without correction for FDR. Spearman’s rank-correlation coefficient was used to correlate gene-expression levels with serum protein levels. Where appropriate, data were transformed using Tukey’s ladder of power transformations to achieve normality, or if this could not be achieved, non-parametric tests were used.

## Results

### Sample characteristics and overall gene expression

Five patients and one control did neither have gene expression nor clinical data, and were excluded from analysis, and several controls did have insufficient gene data available (due to higher than the pre-defined acceptable amount of missing values, as described in the methods section), yielding a final sample of 197 MDD patients and 151 controls. Significant differences of demographic data between control and patient groups emerged for depression severity, CA and BMI, all higher in patients, as expected. Serum IL-6 and hsCRP levels were higher in the MDD group, as a trend effect for hsCRP and significantly for IL-6 (Supplementary Table 1A). No significant differences were found for other variables. More patients with CA experienced medium or high risk for suicide compared to the no-CA group, who had a higher prevalence of no or low risk of CA (*χ*^2^ = 13.36, *P* = 0.004). This relationship is well described in the literature^33^ and taken into account in multivariate analysis of covariance (MANCOVA) models, where SR, CA and their interaction were included. Virtually, all patients used a variety of antidepressants (Supplementary Table 1B and Supplementary Fig. 1).

**Fig. 1:**
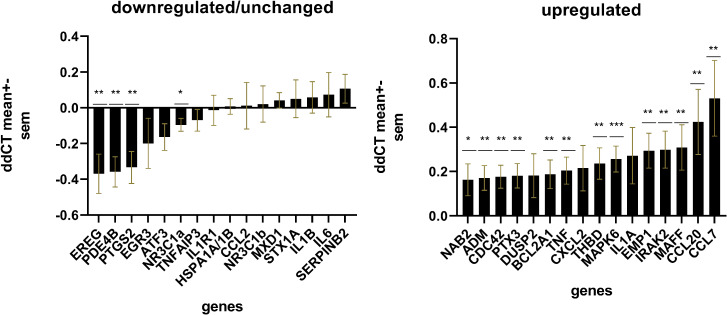
∆∆CT monocyte gene expression in patients with MDD. Results are displayed for MDD patients (*n* = 197) normalised by the housekeeping gene ABL1 and relative to healthy controls without CA (*n* = 95) of each site separately; ΔΔCT values (i.e., logFC) were used for statistical analysis and graphical representations. **a** Unchanged and downregulated genes. **b** Upregulated genes. *P < 0.05; **P < 0.01; ***P < 0.001. *P* values are adjusted for false discovery rate (FDR) with the Benjamini–Hochberg method.

Comparison of genes revealed a general overexpression in MDD patients compared to controls without CA (complete cases, *n* = 95). Seventeen genes reached statistical significance after correction for FDR (Fig. 1). Thirteen genes were significantly up- and four significantly downregulated.

### Primary analyses: bottom-up approach

**Fig. 2:**
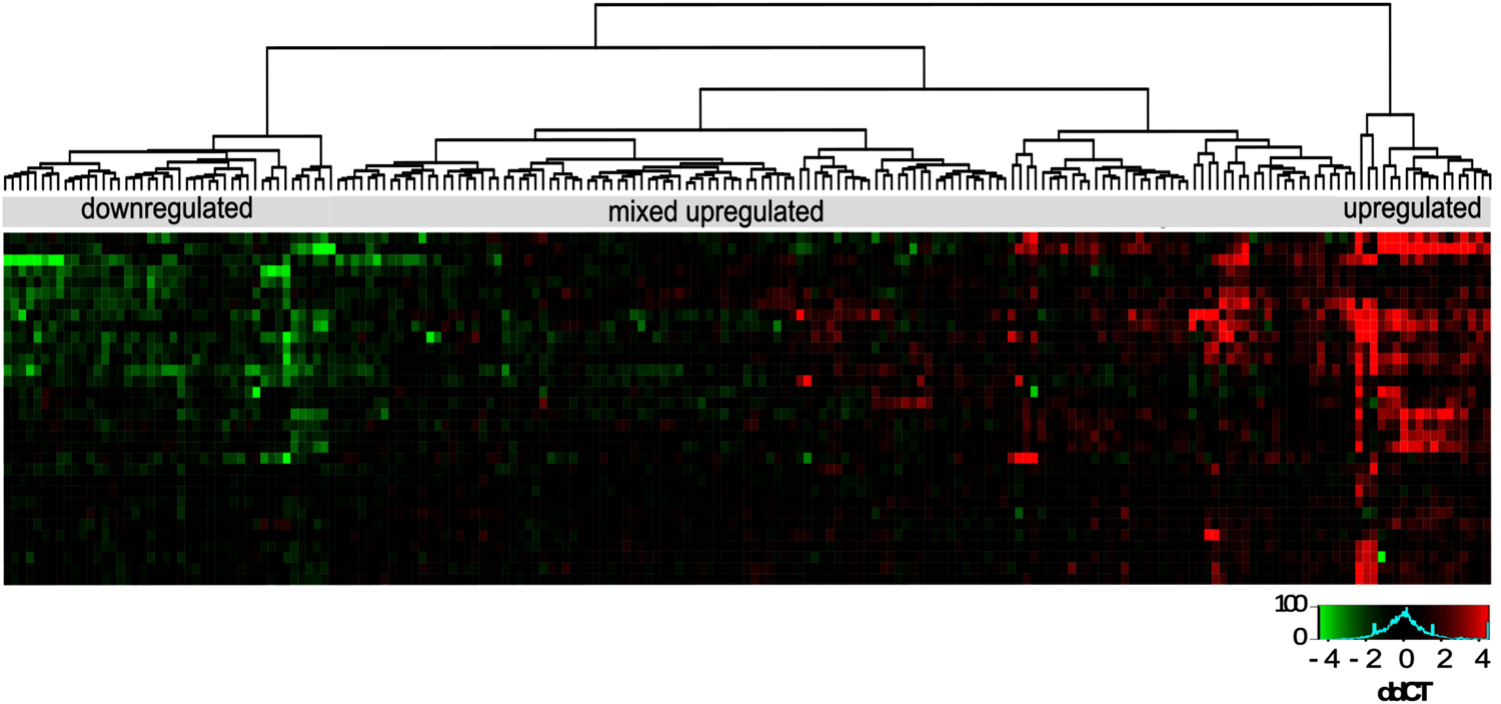
Hierarchical clustering and heatmap of MDD patients based on monocyte gene expression. Three patient groups can be identified: one group with downregulated genes (green, *n* = 44), one group with primarily upregulated and some downregulated genes (*n* = 135) and one with highly upregulated genes (red, *n* = 18). Values represent ΔΔCT gene expression relative to healthy controls without CA.

CA and SR are more frequent in MDD patients with (mixed) upregulated inflammation-related genes in monocytes. A closer examination of gene expression revealed high variability within MDD patients, as hypothesised. Three immunologically distinct groups of patients could be identified through hierarchical clustering: one group showing high upregulation (mean of all genes upregulated, *n* = 18), one overall mixed upregulation (mean of 78% of genes upregulated, *n* = 135) and one clear downregulation of monocyte genes (mean of all genes downregulated, *n* = 44) (Fig. 2). In addition to separate analyses, high and mixed upregulated groups were combined in a separate step, based on similarities of mean gene-expression direction, clinical characteristics and low number of highly upregulated patients (*n* = 18). Subgroups with different gene expression did not differ regarding the distribution of age, sex, BMI, depression severity and medication status. However, more patients in mixed- and combined upregulated compared to the downregulated groups had high SR, and showed higher levels of CA (Table 1). The effects for CTQ sum and SR, but not CA proportions, were significant or showed trends for significance after FDR correction (Supplementary Table 2).

**Table 1:** Demographic differences within immunological subtypes of MDD patients.

Sample characteristics per gene profile											
	Patient cluster							Statistical difference *P* value			
	Upregulated		Mixed upregulated		Downregulated		Overall *P* value	Up versus mixed	Mixed versus down	Up versus down	Merged up versus down
	*n* = 18		*n* = 135		*n* = 44						
Age mean, ±sd	39	15	40	13	36	11	0.173	0.37	0.031	0.2	0.07
BMI mean, ±sd	24	4	25	5	25	5	0.747	0.22	0.41	0.3	0.92
IDSC mean, ±sd	30	10	33	11	31	11	0.362	0.1	0.23	0.24	0.58
CTQ mean, ±sd	41	11	44	15	36	8	0.036*	0.42	0.005**	0.07	0.01*
Sex female (%)	56		67		52			0.5	0.12	1	0.16
SR, high (%)	27		27		9			1	0.032*	0.21	0.03*
Childhood adversity (%)	65		62		44			1	0.092	0.28	0.08

*IDSC* inventory of depressive symptomatology clinican rated, *CTQ* childhood trauma questionnaire short form, *SR* suicide risk. Continuous variables of the three clusters were compared with the Kruskal–Wallis test followed by Dunn’s test. Continuous variables between merged clusters were compared with Wilcoxon–Mann–Whitney test, *P* values are not adjusted for multiple testing. When applying the Benjamini–Hochberg method to correct for multiple testing, CTQ sum score and SR remained significant or showed a trend for significance. Proportions of CA were no longer significant with adjustment for multiple testing. Significance: *p < 0.05, **p < 0.01.

### Primary analyses: top-down approach

Hierarchical clustering of the genes also revealed three clusters of intercorrelating genes. These gene clusters largely overlapped with the previously published gene clusters^16,17^ (Supplementary Fig. 2). Gene cluster 1 roughly comprised various pro-inflammatory cytokine and compound genes (such as IL1B, IL-6, TNF and PTGS2) and regulators of these compounds (ATF3, DUSP2 and MAFF). Gene cluster 2 predominantly related to adhesion, coagulation and chemotactic ability of monocytes (EMP1, STX1A, THBD, CCL2 and CCL7). Genes of the smaller gene cluster 3 predominantly relate to glucocorticoid sensitivity (NR3C1a and HSPA1A/B). These gene clusters are used for the sub-analysis below. It should be noted that the measured genes are not only involved in stimulating but also in blocking inflammation, adhesion, chemotaxis and motility.

**Fig. 3:**
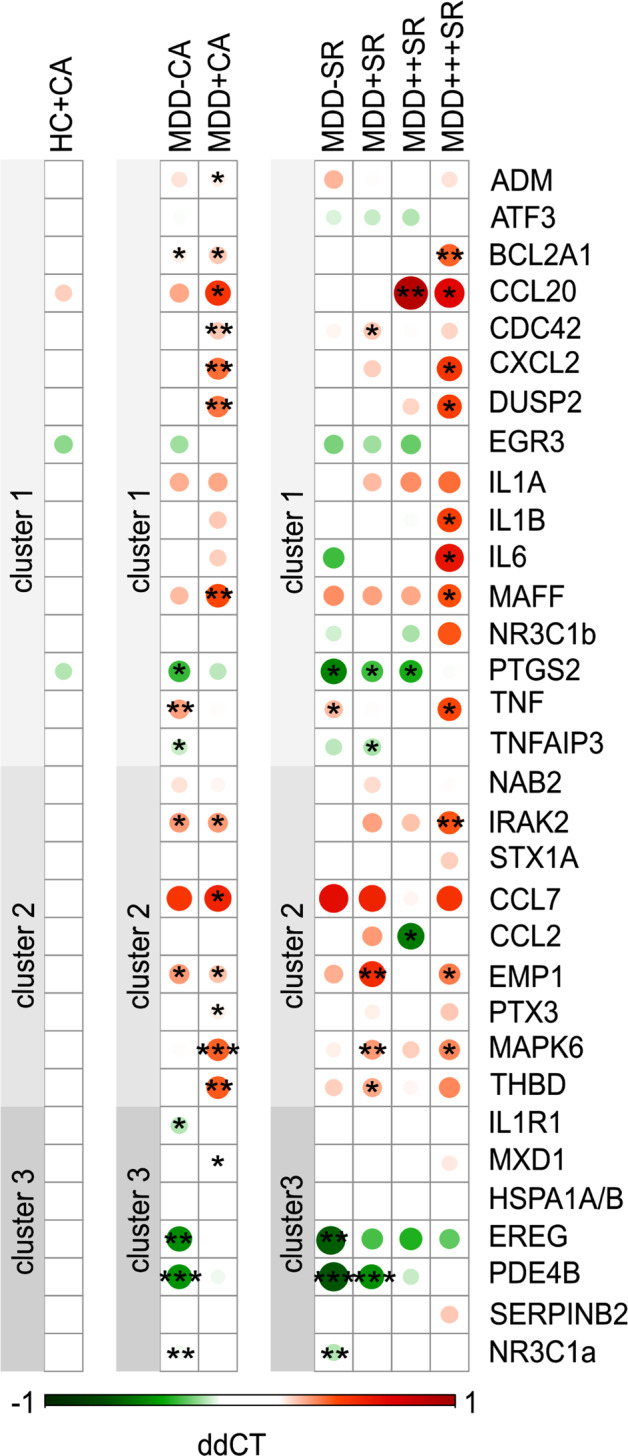
Effects of suicide risk (SR) and childhood adversity (CA). Gene expression in controls and patients compared to healthy controls without CA. Coloured dots represent mean ΔΔCT gene regulation, green colour indicates downregulation and red colour upregulation. Genes that are significantly up- or downregulated are marked with an asterisk (*P < 0.05, **P < 0.01), not corrected for multiple testing. From left to right: healthy controls (HC) with CA (*n* = 56) show no significant alterations compared to healthy controls without CA (reference group, *n* = 95). Within the MDD group, MDD without childhood trauma (*n* = 72) shows predominantly downregulation of genes, with significant effects for genes in cluster 3. MDD with CA (*n* = 103) shows a strong upregulation of genes with significant effects, particularly for MAPK-related genes (CDC42, DUSP2 and MAPK6). MDD patients at high SR (*n* = 42) showed the strongest upregulation, particularly in inflammatory cluster 1, followed by patients with medium SR (*n* = 47), whereas patients without SR (*n* = 41) or with low SR (*n* = 56) showed downregulation of cluster 3 genes. Exact unadjusted and adjusted *P* values can be found in Supplementary Table 3. HC + CA healthy controls with CA, MDD-CA MDD patients without CA, MDD + CA MDD patients with CA, MDD-SR MDD patients without SR, MDD + SR MDD patients with low SR, MDD + + SR MDD patients with medium SR, MDD + + + SR MDD patients with high SR.

To explicitly answer the question whether CA and SR determine differences in clusterwise inflammatory gene expression, we explored monocyte clusterwise inflammation-related gene expression in MDD patients using CA and SR as independent variables. Figure 3 shows the outcomes.

### Effects of SR status on clusterwise monocyte gene expression

In patients at high SR, 88% of genes in cluster 1 or 2 were upregulated, 44% of genes reaching statistical significance. The most significantly upregulated genes still showed a trend for significance after correction for FDR within clusters (Supplementary Table 3).

**Fig. 4:**
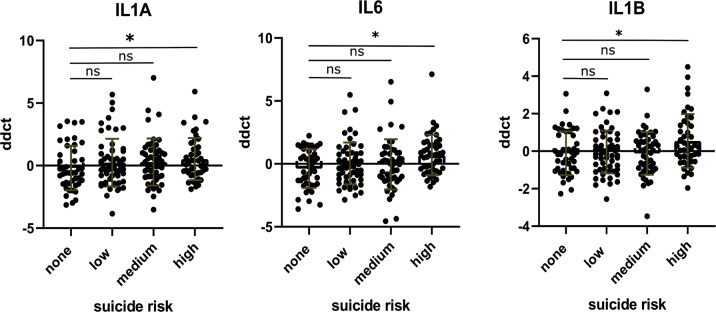
Comparison of significant cluster 1 genes split by suicide risk (SR), MANCOVA, including CA, depression severity, age, sex and BMI, revealed a significant effect for SR on cluster 1 (all imputed datasets pooled *P* = 0.04). *P* values are derived from contrasts of pooled linear models for imputed data, and revealed significant effects for the comparison between no- and high SR groups for (IL-1α: *P* = 0.028, IL-1β: *P* = 0.040 and IL-6: 0.014). The results for IL-1 and IL-6 remained significant after applying robust linear models, while IL-1β was no longer significant.

A significant effect of SR status in the MANCOVA emerged only for gene cluster 1, with relatively stable estimates (approximate *P* value derived from pooling of *F* values from imputed datasets *P* = 0.04). Amongst the upregulated genes, the well-known genes encoding inflammatory interleukins, IL1A, IL1B and IL-6, were significantly higher in the high-risk compared to both healthy controls (all *P* < 0.05) (Fig. 3), and the no-risk group after taking covariates (including CA) into account (Fig. 4). Interestingly, in patients with no SR, hardly any of the cluster 1 and 2 genes were differently expressed compared to the healthy controls. Instead, several cluster 3 genes were significantly downregulated (in particular EREG, PDE4B and the glucocorticoid receptor (GR)-α) (Fig. 3). Although no significant effect emerged during MANCOVA for cluster 3, all cluster 3 genes were downregulated in the no-risk group. Low- and medium-risk groups showed overall intermediate values, and while few genes were differentially expressed compared to controls, they did not statistically differ from the no-SR group when covariates were taken into account.

### Effects of CA on clusterwise monocyte gene expression

In patients who had experienced CA, most cluster 1 and cluster 2 genes were upregulated (93%), out of which 52% significantly, most also after correction for FDR (Supplementary Table 3). This is particularly notable for genes linked to MAP kinase (MAPK) signalling (MAPK6, DUSP2, CDC42 and BCL2A1). No change was observed for glucocorticoid-related genes (Fig. 3). Interestingly, similar to the patient group with no SR, patients without CA showed hardly any significantly upregulated genes (<20%). Instead, they showed downregulated expression of most cluster 3 genes (NR3C1a, PDE4B, EREG and IL1R) versus controls. Yet, when testing the effect of CA in MANCOVA on clusterwise expression, and including covariates, a statistically significant difference did not emerge between patients with and without CA for any of the three intercorrelating clusters (Fig. 3). Likewise, no significant interaction effect emerged between SR and CA. Last but not least, there was no significant change of genes in healthy controls who had experienced CA (*n* = 56) (Fig. 3).

### Covariates

Analysis of covariates showed significant effects of BMI on cluster 1 gene expression with significant levels for TNF expression (*P* = 0.035). Age, sex and depression severity had significant effects on single genes, and were taken into account for the final models, but were not significant for overall clusters as explored by MANCOVA. Medication status as a binary variable showed significant effects, but did not alter the effects for single genes. Yet, due to the high variability of medication usage and low number of unmedicated patients, we did not have sufficient power to investigate these effects with certainty. Additional analyses exploring CA subtypes revealed that cluster 1 was linked to emotional neglect (*P* = <0.001–0.005) and emotional abuse (*P* = 0.011–0.164), cluster 2 gene expression to physical neglect (*P* = 0.002–0.024) and sexual abuse (*P* = 0.011–0340) and cluster 3 gene expression to emotional abuse (*P* < 0.001–0.015) and emotional neglect (0.001–0.067).

### Secondary analyses

*Correlation of monocyte gene expression to serum levels of hsCRP and IL-6*: In MDD patients, serum levels of hsCRP correlated weakly with CCL20, IL1A and PTX3, which showed significance or a trend for significance after correction for multiple testing. Serum levels of IL-6 did not correlate with gene-expression levels of IL-6 (*r* = 0.04, *P* = 0.680). In controls, ADM and TNFAIP3 were weakly correlated with hsCRP after correction for multiple testing (Supplementary Table 4). IL-6 serum levels in controls correlated significantly with expression of NR3C1a, but did not correlate with IL-6 gene expression (Spearman’s *r* = 0.008, *P* = 0.947).

When investigating the effect of SR and childhood trauma groups on serum protein levels while taking into account age, sex, depression severity and BMI for MDD patients, no significant effect emerged for SR on hsCRP (all *P* > 0.50). However, for hsCRP, next to significant main effects for CA and BMI, a significant interaction between those factors emerged (*t* = −2.81, *P* = 0.006). Splitting the groups per BMI (median split, BMI under or above 24.22) showed that for those with a lower BMI, CA significantly correlated with increased hsCRP (*t* = 3.024, *P* = 0.003), while no effect emerged for those in the high BMI group (*t* = −1.48, *P* = 0.145). Of note, CA per se was not associated with a higher BMI (*t* = −0.52, *P* = 0.49). No effect on IL-6 was observed for CA (Wilcoxon rank-sum test *z* = 0.12, *P* = 0.901) or SR (Kruskal–Wallis = 2.41, df = 3, *P* = 0.491).

## Discussion

We here show that with regard to monocyte inflammation-related gene expression, patients with MDD are not uniformly “inflamed”. About 75% of patients showed overall upregulation, whereas about 25% showed general downregulation of monocyte inflammation-related gene-expression patterns. Intriguingly, only CA and SR, but no other sampling variables were different between the up- and downregulated patient groups. Raised inflammatory gene expression in MDD patients was linked to CA and high SR when covariates were taken into account, which contrasts with the overall reduced gene expression in MDD patients without CA and SR. This suggests immunologically discernible phenotypes of depression, which can be linked to clinically meaningful variables.

### Childhood adversity

Exposure to CA was linked to increased monocyte gene expression for patients but not controls. Although no statistically significant discriminative effect was achieved between patients with and without CA, an overall clear upregulation of cluster 1 and 2 genes in the CA-exposed group strongly hints to an immune component in these individuals. Indeed, the link between CA, depression and inflammation is suggestive^23,24,34–36^, and has been confirmed in a recent meta-analysis^37^. The immune system has been proposed as a mediator for the relationship between CA and depression^37,38^. Our results indirectly support this notion, given that immune activation was present in patients, but not healthy controls who had experienced CA. It also suggests that CA per se is not affecting the monocyte inflammatory state, but that other patient-intrinsic mechanisms together with CA confer a vulnerability for depression. In addition, our results show that the previously reported, positive association of CA and hsCRP protein levels^39^ was present in patients with low or normal BMI, but not in those with high BMI. A possible explanation for this effect is that a high BMI leads to an independent increase in serum inflammatory compounds, which may mask the relatively smaller effect of CA in this population. The fact that monocyte gene expression was increased in patients with CA, while the correlation with serum levels of IL-6 and hsCRP was poor, suggests that circulating monocytes contribute, but are not the only driver of inflammatory activation associated with CA.

### Inflammatory monocyte activation and SR

Similar to the patient group with CA exposure, high SR was associated with clear upregulation of genes in clusters 1 and 2. Inflammatory activation of the immune system in patients with SR has recently been described regarding cytokines in serum, cerebrospinal fluid and postmortem studies^40–42^. In particular, levels of not only IL-6^40,43^, but also IL-1β^40^, and a history of hospitalisation with infection have been linked to suicidal behaviour^44,45^. We here observe a similar effect in peripheral monocytes: IL1A, IL1B and IL6 were significantly higher in the high SR group compared to both controls and the no-risk group. Partitioning of patient groups in SR groups may hence explain part of the variability found for inflammatory compounds in MDD. This notion is supported by a recent positron emission tomography study measuring translocator protein (TSPO) in depressed patients. TSPO is usually upregulated in activated (micro-) glia and serves as a proxy for neuroinflammation. The authors found elevated levels in MDD patients at SR, but not those without^46^, further highlighting the distinction between these subgroups of patients, conceivably also in the central nervous system. It should also be noted that serum protein levels of IL-6 poorly correlated with the monocyte gene expression. A poor correlation can be suspected, given that this acute-phase protein’s production also depends on, among others, IL-6 production by other cell types (e.g., T cells, tissue macrophages such as the adipose tissue macrophages), and thus probably reflects the diverse origin and interactions that lead to circulating protein levels of hsCRP and IL-6.

### Immunological pathways of CA and SR in patients

In addition to the well-known interleukins, we found an association of high SR and CA with several MAPK pathway-related genes. The involvement of the MAPK pathway in the pathogenesis of MDD has long been suspected^47^, and accumulating evidence seems to support this notion^48,49^. For instance, DUSP2 has previously been identified to link stress experience and depression pathogenesis: DUSP1 and DUSP2 expression was increased in response to chronic stress in rodents and in the brain of two separate cohorts of depressed patients^49,50^.

Many of the cluster 1 and 2 genes previously picked up by whole-genome analysis, belong to the (atypical) MAPK pathway. It is hence not surprising that the MAPK pathway shows significant results in the present analysis. However, the fact that this pathway is clearly upregulated in clinically distinct patient groups is intriguing and may provide useful hints as to molecular mechanisms distinguishing (immunological) subtypes of depression. Of note, the effect was higher in patients exposed to CA and SR even when depression severity was included in the model, and hence does not merely describe more severe depression. Interestingly, antidepressant actions of regular and rapid-acting antidepressants such as ketamine may depend on MAPK signalling^51,52^: it was shown that MAPK1 levels normalised after antidepressant treatment with fluoxetine^49^, and Reus and colleagues have shown that acute blockade of MAPK signalling abolished the antidepressant effects of ketamine in a rat model^51^. Our results may help to identify more efficacious treatment options, depending on clinical and immune profiles.

Importantly, CA increases the risk for suicidal behaviour^53,54^. Although we here did not observe an interaction effect on specific genes, it is therefore tempting to speculate that CA is linked to suicidal ideation through a long-lasting activation of the monocyte/macrophage system, possibly involving inflammatory compounds and/or the MAPK pathway.

### Trained immunity as the explanatory framework for innate immune changes

The recently accepted theorem of “trained immunity” of the innate cell lineage provides a scientific framework that can explain long-lasting changed equilibrium states of steroid-sensitive and inflammation-related genes in monocytes/macrophages^55^. Monocytes are generally short-lived (i.e., days) in the circulation^56^. While initially thought to be deprived of any form of immune memory (unlike the T- and B-cell systems), it is now accepted that monocytes/macrophages do build up immune memory by epigenetic imprinting after repetitive exposure to stimulating agents^55^. One possibility is therefore that the here-described altered regulation of monocyte gene expression in adult MDD patients could be explained by long-lasting epigenetic changes in monocytes in a process of “trained immunity”.

### Alterations in steroid sensitivity in no-risk groups

As discussed before, CA per se did not affect monocyte inflammatory state in controls, but it did in patients. This suggests that additional mechanisms confer a vulnerability for depression. These mechanisms might be (a combination of) immune-controlling mechanisms^14^ and endocrine signalling, such as HPA-axis abnormalities^17,57^. Indeed, we observed a downregulation of genes in the glucocorticoid-related cluster 3 for patients not exposed to CA and without SR. The genes NR3C1a and HSPA1A/B play important roles in the bidirectional crosstalk between the immune system and the HPA axis^58,59^. While NR3C1a encodes the stimulating subunit of the GR, HSPA1A/B encodes the protein HSP70, which regulates GR function^60^. Downregulation of both may thus alter glucocorticoid sensitivity. Indeed, an altered HPA axis, high levels of glucocorticoids and immune/inflammatory factors have been conceived as offenders in the pathophysiology of MDD^59^. Patients without SR or not exposed to CA may thus constitute another subtype of depression, characterised by different states of sensitivity for glucocorticoids. Whether these different states of expression of steroid sensitivity-related genes do lead to higher or lower sensitivity for glucocorticoids and impact inflammatory activation, needs further exploration in functional studies.

### Consideration for treatment strategies

The subdivision of MDD in immunologically discernible phenotypes is important in light of current discussions on anti-inflammatory agents to treat patients with MDD^9,61^. Our findings suggest that this practice requires caution: patients with a downregulated monocyte phenotype may not benefit, or even incur harm from further immunological suppression. Stratification of patients based on peripheral markers has proved useful before^12,62^. Our results support this notion and urge for further studies to stratify patients in “inflamed” and “not inflamed/supressed”, when studying the effect of regular antidepressants and anti-inflammatory drugs. When designing future studies, the effect of CA and SR on inflammatory activation should be recognised and accounted for during potential treatment stratification, as to lead to more successful treatment selection. Ideally, future study designs would use a longitudinal design in order to assess the relationship between CA and SR more extensively.

### Limitations

Firstly, the nature of this study is associative, and although we can probably assume temporal precedence of CA to gene signature overexpression, we cannot assume a causal relationship between the two. We here used an arbitrary yet previously validated cut-off of exposure to CA as a binary variable; however, the grade of exposure to CA may certainly impact depression pathogenesis. Furthermore, as indicated before, we used monocyte inflammation-related genes preselected in earlier and previously published findings and validation studies. MDD patients and controls with and without CA may very well have other monocyte gene-expression changes not directly related to inflammation and not reflected in the here-described set of preselected genes: others also showed the effects of immediate stress exposure on a few of the same inflammation-related monocyte genes as described here (HSPA1A, STX1A and IL-6), and on genes involved in other pathways^63^. In addition, we here identified a mixed upregulated cluster, which showed upregulation in ~78% of genes. However, several of the genes did not show a strong pattern of upregulation, and it could therefore also be argued that these patients show no alteration. An important limitation is also that inflammatory gene expression in circulating monocytes does not correspond to peripheral protein levels of the inflammatory compounds. We here found no association of SR with serum protein levels of IL-6 and with hsCRP, this may indicate that our findings are specific to certain cellular compartments of the innate immune system and not generalisable to the overall immune state. Lastly, the results comparing no- and high SR groups were not corrected for FDR. Although we had a large group of MDD patients, subdivision based on SR yielded comparatively small groups. Our analyses should be replicated with particular focus on SR.

## Conclusion

Our data suggest immunologically discernible phenotypes of depression: while most patients indeed show signs of inflammatory activation, a considerable number of patients show downregulated gene expression of peripheral monocytes. Interestingly, both CA and high SR were linked to increased expression of genes encoding inflammatory compounds. Although no causal assumption can be made based on the presented cross-sectional data, these results highlight the importance of determining clinical variables when assessing biological phenotypes of depression. Future studies should replicate the results in sufficiently large groups, and use other markers of inflammation (e.g., cytokines). If replicated, our results warrant stratification of patient groups by a combination of clinical and immunological characteristics for a more personalised treatment approach.

## Supplementary information

Supplementary Notes

Supplementary Table 1

Supplementary Figure 1

Supplementary Table 2

Supplementary Figure 2

Supplementary Table 3

Supplementary Table 4

## Data Availability

The R code is available from the first author upon request.
