# Supplementary material for: Childhood trauma, suicide risk and inflammatory phenotypes of depression: insights from monocyte gene expression
Source: Transl Psychiatry. 2020 Aug 24;10:296. doi: 10.1038/s41398-020-00979-z (PMC7445278; doi:10.1038/s41398-020-00979-z)
Supplement: Supplementary file 1 — Supplementary Notes [file 41398_2020_979_MOESM1_ESM.doc]

**Supplementary information S1. Explanation of gene selection procedure.**

The here used genes were first established in finding studies using whole genome Affymetrix analyses on CD14-purified monocytes of patients with bipolar disorder, thyroid autoimmune disease, schizophrenia and type 1 and type 2 diabetes ^1-4^. In these 5 relatively small finding studies, carried out about a decade ago, genes were strictly corrected for False Discovery Rates (see publications). Genes for further studies were selected on the basis of statistically significant top over and under expression (over 3 fold up or down regulation to healthy control monocyte values) and an obvious relation to inflammation-related processes in Ingenuity analyses.

The altered expressions of the selected “top genes” (on average around 40-50 genes per study) were validated in various confirmation studies using Q-PCR techniques and using larger cohorts of patients (n=40-50). These patients were characterized by low grade inflammation, i.e. bipolar patients ^1, 4, 5^, relatives of bipolar patients ^6, 7^, post-partum psychosis patients ^8^, schizophrenia patients^4^, major depressed patients ^9-11^, type 1 diabetes patients ^2, 12^, type 2 diabetes patients ^13, 14^ and autoimmune thyroiditis patients^3^. Using hierarchical clustering in the confirmation studies it became apparent that almost always the genes were expressed in 2-3 main clusters of strongly inter-correlating genes (as in this study)

Using ingenuity analysis on the gene signature and its sub-clusters, it additionally became apparent that the gene signature was composed of both positive and negative regulators of several important inflammation-related processes, such as the production of pro-inflammatory cytokines (mainly occurring in a sub-cluster, called sub-cluster 1 in the afore-mentioned studies) and of chemotaxis, motility and adhesion (mainly occurring in a sub-cluster, called sub-cluster 2 in the afore-mentioned studies).

The outcome of above studies resulted in the selection of a panel of inflammation related genes (around 40-50), which were prominently altered expressed in the various confirmation studies. We used these genes in the MOODINFLAME studies. We here analyse genes that were measured in most patients in all 3 clinical centers and where less than 30% of gene expression data was missing per site.

**References**

1. Padmos RC. *et al*. A discriminating messenger RNA signature for bipolar disorder formed by an aberrant expression of inflammatory genes in monocytes. *Archives of general psychiatry* 2008; **65**(4)**:** 395-407.

2. Padmos RC. *et al*. Distinct monocyte gene-expression profiles in autoimmune diabetes. *Diabetes* 2008; **57**(10)**:** 2768-2773.

3. van der Heul-Nieuwenhuijsen L. *et al*. An inflammatory gene-expression fingerprint in monocytes of autoimmune thyroid disease patients. *The Journal of clinical endocrinology and metabolism* 2010; **95**(4)**:** 1962-1971.

4. Drexhage RC. *et al*. Inflammatory gene expression in monocytes of patients with schizophrenia: Overlap and difference with bipolar disorder. A study in naturalistically treated patients. *International Journal of Neuropsychopharmacology* 2010; **13**(10)**:** 1369-1381.

5. Vogels RJ, Koenders MA, van Rossum EF, Spijker AT, Drexhage HA. T Cell Deficits and Overexpression of Hepatocyte Growth Factor in Anti-inflammatory Circulating Monocytes of Middle-Aged Patients with Bipolar Disorder Characterized by a High Prevalence of the Metabolic Syndrome. *Frontiers in psychiatry* 2017; **8:** 34.

6. Padmos RC. *et al*. Genetic and environmental influences on pro-inflammatory monocytes in bipolar disorder: a twin study. *Archives of general psychiatry* 2009; **66**(9)**:** 957-965.

7. Mesman E. *et al*. Monocyte activation, brain-derived neurotrophic factor (BDNF), and S100B in bipolar offspring: a follow-up study from adolescence into adulthood. *Bipolar disorders* 2015; **17**(1)**:** 39-49.

8. Bergink V. *et al*. Immune system dysregulation in first-onset postpartum psychosis. *Biological psychiatry* 2013; **73**(10)**:** 1000-1007.

9. Carvalho L. *et al*. Inflammatory activation is associated with a reduced glucocorticoid receptor alpha/beta expression ratio in monocytes of inpatients with melancholic major depressive disorder. *Translational psychiatry* 2014; **4**(1)**:** e344-e344.

10. Grosse L. *et al*. Clinical characteristics of inflammation-associated depression: Monocyte gene expression is age-related in major depressive disorder. *Brain, behavior, and immunity* 2015; **44:** 48-56.

11. Weigelt K *et al*. TREM-1 and DAP12 expression in monocytes of patients with severe psychiatric disorders. EGR3, ATF3 and PU. 1 as important transcription factors. *Brain, behavior, and immunity* 2011; **25**(6)**:** 1162-1169.

12. Beyan H. *et al*. Monocyte gene-expression profiles associated with childhood-onset type 1 diabetes and disease risk: a study of identical twins. *Diabetes* 2010; **59**(7)**:** 1751-1755.

13. Baldeón RL. *et al*. Type 2 Diabetes Monocyte MicroRNA and mRNA Expression: Dyslipidemia Associates with Increased Differentiation-Related Genes but Not Inflammatory Activation. *PLoS One* 2015; **10**(6)**:** e0129421.

14. Baldeón L. *et al*. Study on inflammation-related genes and microRNAs, with special emphasis on the vascular repair factor HGF and miR-574-3p, in monocytes and serum of patients with T2D. *Diabetology & metabolic syndrome* 2016; **8**(1)**:** 6.
