# Supplementary figures and images for: Childhood trauma, suicide risk and inflammatory phenotypes of depression: insights from monocyte gene expression
Source: Transl Psychiatry. 2020 Aug 24;10:296. doi: 10.1038/s41398-020-00979-z (PMC7445278; doi:10.1038/s41398-020-00979-z)

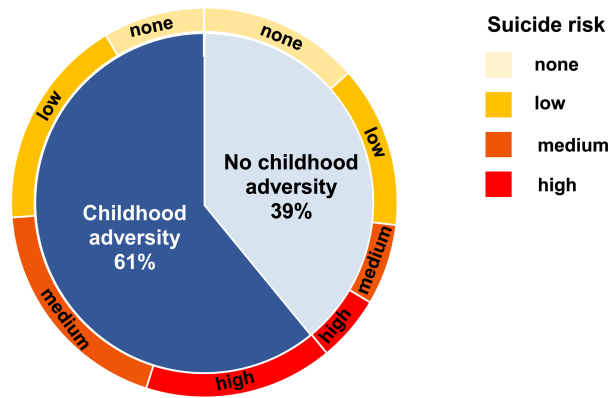

Supplement: Supplementary file 3 — Supplementary Figure 1 [file 41398_2020_979_MOESM3_ESM.pdf]

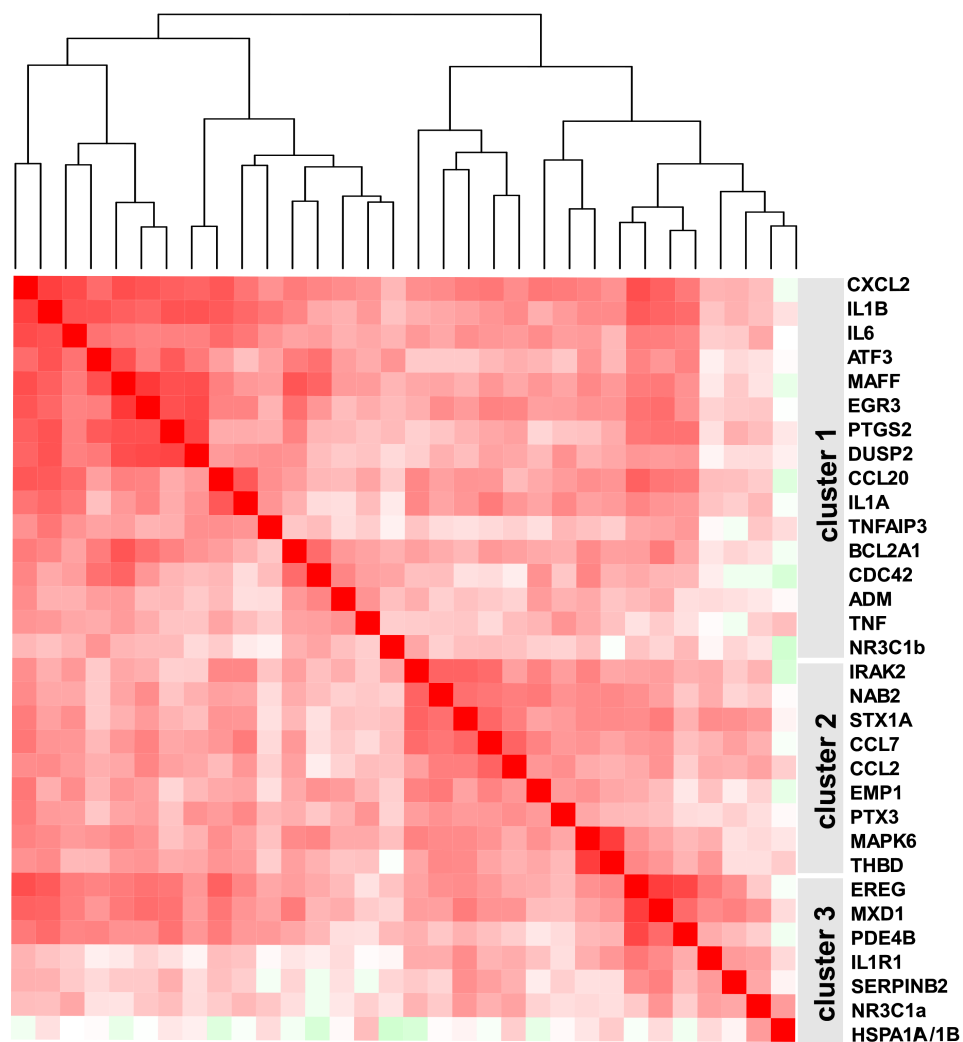

Supplement: Supplementary file 5 — Supplementary Figure 2 [file 41398_2020_979_MOESM5_ESM.pdf]
